# Evaluating the effectiveness of a school-based mental health literacy intervention from a comprehensive demographic and social-cognitive perspective

**DOI:** 10.1038/s41598-024-56682-2

**Published:** 2024-03-11

**Authors:** Yifeng Wei, Li Sha, Robert McWeeny, Rav Johal, Constance Easton, Andrew Baxter, Bo Cao, Andrew Greenshaw, Wendy Carr

**Affiliations:** 1 Department of Psychiatry, University of Alberta, 1E1 Walter Mackenzie Health Sciences Centre (WMC), 8440 112 St NW, Edmonton, AB T6G 2B7 Canada; 2 Department of Psychiatry, University of Alberta, Edmonton, AB Canada; 3 Richmond School District No. 38, Richmond, BC Canada; 4 Alberta Health Services, Calgary, AB Canada; 5 Faculty of Education, University of British Columbia, Vancouver, BC Canada

**Keywords:** Risk factors, Signs and symptoms

## Abstract

Childhood and adolescence are a critical period for the onset of mental and neurodevelopmental disorders and a time when many can be first identified. Research demonstrates that mental health literacy applied in school settings may be an effective approach to address these challenges. In contrast to many existing studies conducted in multicultural and multilingual settings that treated subjects’ language as a demographic feature, the present study recognizes English proficiency as a social-cognitive factor and views the school-based mental health literacy (MHL) intervention as a learning process. The present study aimed to assess the effectiveness of school-based mental health literacy intervention and explore how ethnicity and English proficiency as a social-cognitive factor, as a modified, rather than a fixed variable, impacted the intervention outcomes. Grade 9 students (*n* = 240) from schools in West Canada with diverse social/cultural background received the intervention in the classroom delivered by trained teachers and completed the pre-test and post-test over a 6-month period. The intervention was effective in improving knowledge and help-seeking attitudes among all students. Non-Chinese and native English-speaking students performed the best on all outcomes. Gender demonstrated an association with changes in stigma, stress and wellbeing. English proficiency was linked to knowledge acquisition, while ethnicity was connected to changes of attitude-related outcomes. These findings deepened our understanding of how demographic and social-cognitive factors underlie changes in mental health literacy outcomes, which will facilitate the development of mental health literacy interventions for diverse student populations.

## Introduction

Most mental disorders first develop during adolescence (ages 12–25)^1^. Adaptive plasticity marks adolescence as a critical window of opportunity to improve mental health outcomes through promotion of resilience, recovery, and healthy development^2^. Mental health literacy (MHL) approaches are considered foundational for mental health promotion, prevention, and care and are well-validated in adolescent mental health contexts^3^. School-based MHL interventions address domains of MHL: knowledge and skills to obtain and maintain positive mental health, stigma about mental disorders, knowledge about mental illness and related treatment, help-seeking intentions and behaviours, and/or stress and enhancing resilience^3–7^. Freţian et al.^6^ (2021) suggested that school-based MHL interventions instigate greater knowledge acquisition than attitude change among students, which is supported by a meta-analysis^8^ that revealed most MHL interventions showed significant knowledge improvement, and minor impacts on stigma and help-seeking. Further research should more closely examine the contexts of efficacy for these interventions^9^. This would involve greater articulation of the mechanisms, contexts and outcomes of MHL interventions. The present study bridges this gap by examining how gender, ethnicity, English proficiency, and previous MHL experience independently and/or interactively predict changes of key MHL components: knowledge, stigma, help-seeking attitudes, and general health outcomes of stress and wellbeing in a school-based MHL intervention.

Development and implementation of MHL interventions is context-dependent^3^. Specific mechanisms enable interventions to be contextually sensitive, thus generating contextually-informed outcomes^9^. In school-based interventions, these mechanisms comprise implementation factors such as school personnel, pedagogical approaches, assessment tools, and more. Attention to these mechanisms can impact how contextual factors (i.e., age, gender, ethnicity, and location) are represented in an intervention’s outcomes.

Many prior studies have focused on associations between MHL outcomes and sociodemographic characteristics^5,10,11^, including associations between gender, age, ethnicity, family background, knowledge and help-seeking intentions^12–14^.

Ethnicity is distinctly represented in the MHL literature as an area of interest. Clough et al.^13^ found significant differences in knowledge and help-seeking attitudes between international and domestic students in Australia. Wang et al.^15^ found that Asian-American students were less likely than Caucasian counterparts to seek help for mental health concerns. Additionally, Kim and Lee^5^ found that, among Asian-Americans, higher levels of English proficiency were significantly associated with help-seeking likelihood. In addition, these authors noted that English proficiency combined with American acculturation was positively associated with seeking help from mental health professionals. Li et al.^16^ put acculturation, ethnicity, and English proficiency into a regression model that implicated ethnicity as the only significant predictor for help-seeking attitudes among Chinese international students in the United States. Conversely, Choi et al.^17^ assert that English proficiency is the most-significant predictor of MHL among Korean-American women compared to social support and baseline depression levels. In sum, these findings suggest that MHL differences between students may be independently or interactively predicted by ethnicity and English proficiency^18^. Tailored analysis is needed to explore the independent and/or interactive roles of ethnic background and English proficiency on MHL outcomes.

According to the learning as conceptual change model^19^, learning not only comprises acquisition of knowledge, but also changes of attitude, emotions, and behaviors. This is consistent with MHL outcome improvements, which involves shifts in cognition, attitudes, and behaviours^3^. The sociocultural theory of learning^20^ suggests that cognition is socially constructed, context-dependent, and supported by language. In particular, language is an essential component of learning for English first-language and second-language learners; for the latter, there are myriad factors that impact English language acquisition and comprehension, including literacy (in first- and/or second languages), years of learning English, academic support and scaffolding, and more^21^. As such, the present study reconceptualizes English proficiency as a social-cognitive factor, rather than a demographic feature as observed in some existing studies^16,17^.

Past research in multicultural communities suggests that English-language is conceptually intertwined with demographic factors such as culture and ethnicity^16,17^. However, ethnic background is innately unchangeable^22^, while English proficiency is cognitively malleable. One could postulate that enhancing English proficiency may impact MHL outcomes. This further rationalizes our reconceptualization of students’ English proficiency as a social-cognitive factor rather than an unchangeable demographic feature^22^. This is likely to offer a more comprehensive understanding of the interplay between language and MHL outcomes, leading to tailored interventions in the future. No prior research has observed potential impact of English proficiency on MHL outcomes from this perspective. This rationale forms the first hypothesis of our study that English proficiency, as a social-cognitive variable, is the most significant predictor for mental health knowledge acquisition in a school-based MHL intervention for adolescents.

The second hypothesis is that ethnicity plays an additional and distinct role in MHL outcomes and is more associated with attitude change than knowledge. Our study asked students to self-identify whether they considered themselves to be Chinese, thus establishing ethnicity as a subjective variable. This hypothesis can be rationalized by theories of attitude formation towards health behaviors, wherein attitude formation is based on cognitive, affective, and behavioural processes^23^. Cultural socialization utilizes these processes to uphold ethnically specific attitudes and values. For instance, emotional self-control and shame are cultural characteristics of students with Chinese heritage^24,25^ and are more of a barrier to professional help-seeking in this population than European counterparts^26^.Wong et al.'s study^27^ uncovered variations in attitudes toward seeking mental health professional assistance among Chinese individuals from different contexts. Specifically, the study found that Mainland Chinese exhibit a lower willingness to seek counseling professional help compared to both Chinese Australians and Hong Kong Chinese. Notably, data collected from a group of East Asian immigrants, including Chinese individuals in the US, indicated a positive association between English proficiency and the likelihood to seek professional psychological help^28^. These findings indicate that ethnic background is a key differentiator in attitudes toward mental health services between Chinese students and host students in English-speaking countries like the US and Australia. Meanwhile, within Chinese communities, English proficiency emerges as the most significant factor contributing to such differences. The former underscores the significant influence of cultural factors on the disparities in help-seeking behavior among individuals with diverse ethnic backgrounds. It logically implies that assimilating culturally into English-speaking mainstream societies is expected to be an effective approach to narrow this gap. This suggests the pivotal role of English proficiency in differentiating help-seeking attitudes within Chinese communities.

The third hypothesis proposes an interaction effect of ethnicity and English proficiency on the intervention effectiveness. One’s experiences and beliefs are encoded in a native-language context and may be retrieved differently when communicating with a non-native language speaker^29^. These authors found that medical judgments and perceptions among Chinese-English bilingual individuals are influenced by both content and language of communication. This study revealed that Chinese-English bilingual individuals may perceive medical conditions as easier to cure, less painful, and less distressing when communicated in English rather than in Chinese (the native language). This language effect was strongest among participants with high proficiencies in both Chinese and English. These findings suggest that language and culture are closely intertwined in conveyance and perception of cultural norms^29^.

The present study is the first attempt to explore whether and how students’ ethnic background and English proficiency independently and interactively predict the improvement of mental health knowledge, stigma, help-seeking attitudes, stress, and wellbeing between pre- and post-test in a school-based MHL intervention, with prior MHL experience treated as a covariate. Viewing this intervention as a learning process for students is the basic assumption of our research hypotheses, which we tested by addressing the following research questions:

1. Is this school-based MHL intervention effective for all students regardless of demographic differences (gender, ethnicity) and social-cognitive factors (English-proficiency, previous MHL experience) in each of the outcomes?
2. When students are regrouped according to binary variables (male vs. female, Chinese vs. non-Chinese, English as a Second Language (ESL) vs. non-ESL, previous MHL experience vs. no previous MHL experience), which group benefited most from the intervention and in which MHL outcomes?
3. Did individuals’ ethnic background and English proficiency independently and/or interactively predict changes in MHL and general-health outcomes between pre- and post-test after controlling for pre-test measures, and if so, how?

## Methods

### Intervention, participants and procedure

We embedded a MHL intervention, the teaching of the *Mental Health & High School Curriculum Guide* (the Guide) in Grade 9 classrooms, involving 440 students (ages 14 to 16) in an urban school district that hosts the largest proportion of students with Chinese backgrounds in British Columbia, Canada. This school district has 11 secondary schools eligible for this study and 9 secondary schools volunteered to participate in this study. Out of the total 440 students, 240 actively participated in both the pre- and post-test, forming the subjects for the subsequent analysis. The details of demographic features of those 240 students are presented in Table 1. The Guide consists of six modules: stigma of mental illness; understanding mental health and mental illness, information on specific mental illnesses (e.g., anxiety, attention deficit hyperactivity disorder, bipolar, depression, eating disorders, schizophrenia, obsessive compulsive disorder, and post-traumatic stress disorder); experiences of mental illness; seeking help and finding support; and the importance of positive mental health. Each module is designed for teachers’ ease-of-use, with pre-designed teaching content and classroom strategies, learning objectives, background information, classroom activities, and supplementary resources. Discussion of mental illnesses in module 3 includes anxiety, attention deficit hyperactivity disorder, bipolar, depression, eating disorders, schizophrenia, obsessive compulsive disorder, and post-traumatic stress disorder. Student in Module 3 learn to recognize that mental illnesses are associated with changes in usual brain functions; and gain a better understanding of the symptoms, causes, treatments and other supports for specific mental illnesses that commonly arise during adolescence. When teaching, students are divided into a few groups with each group assigned a specific mental disorder for them to dive into details and present in the classroom. Five classroom ready activities are pre-designed for teachers to use in the classroom, such as PowerPoint presentations, and discussion questions. Teachers received training from the school district core trainer on how to deliver the modules and subsequently taught the Guide in the Healthy Living classroom over the course of the 2021–2022 academic year. Each module features pre-designed classroom activities, with embedded core content that teachers have to teach specified in each module, and supplementary resources that teachers can choose to use. This allows teachers to teach the content using their familiar pedagogies. The fidelity lies in the content, not in the delivery methods because no two teachers teach the same subject the same way. Each module engaged students using PowerPoint presentations, discussions, video clips, role plays, and group exercises. At the beginning of Module 1, classroom teachers collected baseline data with a paper questionnaire after consent was obtained from students’ parents or guardians. Teachers collected the post-test data using the same questionnaire at the end of Module 6. Ethics approval including passive consent was granted by the Health Research Ethics Board of the University of Alberta (#Pro00115498). All research was performed in accordance with relevant guidelines/regulations, and passive consent was obtained from all participants and their legal guardians.

**Table 1:** Sociodemographic Characteristics of Participants.

	N	%
Gender		
Female	132	56.2
Male	103	43.8
English level		
ESL	75	31.2
Non-ESL	165	68.8
Ethnicity		
Chinese	133	55.4
Non-Chinese	107	44.6
Previous MHL experience		
Having	110	45.8
Not having	130	54.2

### Measures

*Mental health knowledge* was measured with a 30-item questionnaire based on the content of the Guide resource and required participants to choose from one of three options: ‘true,’ false’ or ‘do not know.’ Each correct answer received one point for a total possible score of 30. It is a well validated instrument addressing 5 topics; brain functions, basic facts about mental health and mental illness, information about specific mental disorders, consequences of untreated mental illness, and mental-illness treatments^30^.

*Stigma* was measured as a total score across 12 seven-point Likert scale statements. Higher scores represented more positive attitudes, indicating lower stigma. Each participant received a potential total score between 7 and 84. The measure demonstrated strong reliability and validity in previous studies^30^.

*Help-seeking intentions* was measured as a total score across 5 seven-point Likert scale statements with each response assigned a value between 1 and 7. Higher scores indicate more positive attitudes. Each participant received a potential total score between 5 and 35. A previous study indicated strong validity of this one-factor subscale^30^.

*Perceived stress level* was measured as a total score across 10 five-point Likert scale statements with each response assigned a value between 0 and 4. Higher scores indicate more perceived stress. Each participant received a potential total score between 0 and 40. This measure has demonstrated strong reliability and validity in previous studies^31^.

*General well-being* was measured as a total score across 5 seven-point Likert scale statements with each response assigned a value between 1 and 7. Higher scores indicate better health outcomes. Each participant will receive a potential total score between 5 and 35. A previous study has demonstrated strong reliability and validity of the general well-being measure^32^.

*English proficiency* was determined by categorizing students into either the ESL group or non-ESL group through their self-response to the question “Would you like to self-identify as an English as a Second Language Learner?”.

*Pre-MHL* was assessed by asking students this question “Have you received any courses/sessions on mental health?”.

### Data analysis

Data screening was done prior to subsequent analyses to identify outliers. The z-scores of the values of all of the 10 continuous MHL variables (e.g., Pre-Knowledge, Post-Helpseeking) respectively from the pre-and post-test were computed. Only a few of those with absolute z-scores > 3.08 (*p* < 0.001) were treated as univariate outliers and replaced with trimmed scores equal to one raw scale unit greater than the highest score falling below the criterion z-value of 3.08^33^. In the meantime, there were no multivariate outliers defined at the 0.001 significance level for Mahalanobis distance^33^.

In order to address the first and second research questions, a set of paired t-tests was used to investigate whether the mean differences are statistically detectable in each of the five MHL components between the pre-test and post-test, with respect to the four binary variables. This multiple comparison presumably gives rise to familywise Type I error that is controlled in the present study by adopting the false discovery rate (FDR) method^34^. This approach is less conservative and greater efficacy, compared to other methods like the Bonferroni correction^35^. Specifically, we conducted five separate paired t-tests on five MHL outcomes (dependent variables), such as knowledge, with each test corresponding to a different categorical variable, such as gender. The FDR formula is given by ${p}_{i}$ = α * i / n, where ${p}_{i}$ represents the adjusted p value for the ${i}^{th}$ dependent variable with the ${i}^{th}$ smallest pre-adjusted p value. Here, we set *α* = 0.05 as the pre-set *p*-value, *n* = 5, and i takes values 1, 2, 3, 4, 5 because of the five separate paired t-tests. Taking the set of paired t-tests among the students with both non-Chinese and non-ESL as an example, the five pre-adjusted *p*-values was sorted in ascending order: < 0.001, 0.021, 0.026, 0.039, 0.112. According to the FDR formula, ${p}_{1}$=0.001 < 1*0.05/5 = 0.01, ${p}_{2}$<0.021 = 2*0.05/5 = 0.02, ${p}_{3}$=0.026 < 3*0.05/5 = 0.03, ${p}_{4}$=0.039 = 4*0.05/5 = 0.04, and ${p}_{5}$=0.112 > 0.05. Consequently, only the first four *p*-values require adjustment according to the Benjamini–Hochberg approach. All the *p*-values reported in Table 4, shown below, have undergone this adjustment.

The third research question was addressed through the use of five multiple regression models. In these models, the post-test measures, such as post-knowledge, served as dependent variables. The independent predictors encompassed corresponding pre-test measures, like pre-knowledge, along with demographic factors such as gender, ethnicity, English proficiency, and prior experience in mental health. As the first step, we included the interaction between English proficiency and ethnicity in each model, computed as their product, as the current study relatively highlights the effects of these two demographic variables on the MHL outcomes. No significant interaction effect was observed on any MHL outcome. As a result, for the second step, we conducted additional regression analyses excluding the interaction, presented in Table 5. All VIF values are below 2, indicating that the assumption of multicollinearity is satisfied.

We performed two-way ANCOVA to merely investigate the interaction effect of students' ethnicity and English proficiency on the post-measures (e.g., post-knowledge) of the five MHL components, using the corresponding pre-test measures (e.g., pre-knowledge) as covariates. Levene's test was used to evaluate the assumption of homogeneity of variances.

### Ethical approval

This study was approved by the Health Research Ethics Board of the University of Alberta (#Pro00115498).

## Results

All measures showed good internal reliability, *α* > 0.70 (Table 2), indicating acceptable reliability^36^. The Pearson correlations (Table 3) indicate acceptable concurrent validity of the measures, for the relationships between the 10 variables were consistent with their theoretical hypothesis, for example, a negative correlation between stress and wellbeing. Meanwhile, most of them were statistically significant and moderate in size (the largest one is 0.71, and most are below 0.40), an acceptable level of multicollinearity of these measures for the subsequent multivariate analyses^33^.

**Table 2:** Mean Scores and Alphas of Knowledge, Stigma, Help-Seeking, Stress, and Wellbeing in the Pre-and Post-Test.

Variables	N	Min	Max	Mean	STD	Skewness	Kurtosis	alpha
PreKnowledge	240	3	14	10.08	2.40	−.70	.41	.76
PostKnowledge	240	5	27	16.15	4.56	.21	−.18	.80
PreStigma	240	40	84	66.33	10.30	−.50	−.52	.84
PostStigma	240	40	84	66.97	10.18	−.44	−.47	.84
PreHelpseeking	237	14	35	25.52	4.62	.02	−.01	.74
PostHelpseeking	239	5	35	26.04	4.90	−.41	.97	.77
PreStress	233	1	40	19.74	7.58	.15	−.24	.87
PostStress	234	0	40	19.77	7.49	.02	−.19	.88
PreWellbeing	232	5	30	17.69	5.18	.13	−.06	.87
PostWellbeing	234	5	30	17.71	5.33	.12	−.36	.86

**Table 3:** Zero-Order Pearson Correlations Between Knowledge, Stigma, Help-Seeking, Stress, and Wellbeing from the Pre-and Post-Test.

	1	2	3	4	5	6	7	8	9
1.PreKnowledge									
2.PostKnowledge	.52**								
3.PreStigma	.28**	.33**							
4.PostStigma	.20**	.33**	.65**						
5.PreHelpseeking	.13*	.03	.15*	.09					
6.PostHelpseeking	.04	.08	.05	.16*	.48**				
7.PreStress	.19**	.20**	.28**	.25**	−.19*	−.22**			
8.PostStress	.12	.10	.14**	.13*	−.13	−.28**	.71**		
9.PreWellbeing	−.04	−.13*	−.14*	−.14*	.34**	.34**	−.65**	−.52**	
10.PostWellbeing	−.03	−.08	−.12	−12	.22**	.22**	−.51**	−.64**	.58**

* < .05, ** < .01.

To address the first research question, the sample was divided into two sub-groups by four binary variables: gender (male vs. female), ethnicity (Chinese vs. non-Chinese), previous MHL experience (present vs. not present), and English proficiency (ESL vs. non-ESL) for sub-group analysis (Tables 4). Six students self-identified as an “other” sex and, due to the small sample, were not included in the gender analysis. The paired T-tests revealed several key findings organized by the MHL variables, as presented in Table 4.

**Table 4:** The Significant Mean Differences in the MHL Variables Between Pre- and Post-Test: Gender, Ethnicity, English Proficiency, and Previous MHL Experience.

Demographic Group	N	T	Mean Difference (S.E.)	Cohen’s d
Knowledge Acquisition				
All students	240	24.11	6.08** (.25)	1.56
Male***	103	17.67	5.62** (.34)	1.64
Female	132	17.12	6.30** (.37)	1.49
Chinese	133	17.29	5.68** (.33)	1.50
Non-Chinese	107	16.95	6.56**(.39)	1.64
ESL	75	13.78	4.87** (.35)	1.59
Non-ESL	165	20.64	6.62**(.32)	1.61
Pre-experience	130	18.36	6.46** (.35)	1.61
No pre-experience	110	15.78	5.62** (.36)	1.51
Non-Chinese and Non-ESL	86	14.78	6.84**(.46)	1.59
Attitude Improvement				
Non-Chinese	107	1.68	1.45*(.86)	.16
Non-Chinese and Non-ESL	86	1.97	1.84*(.93)	.21
Help-seeking Improvement				
All students	236	1.68	.53* (.32)	.11
Male	101	2.14	1.07* (.50)	.21
Non-Chinese	107	2.09	.89*(.43)	.20
Non-ESL	163	2.04	.69*(.34)	.16
No pre-experience	107	2.25	1.01* (.44)	.22
Non-Chinese and Non-ESL	86	2.03	.85*(.41)	.22
Stress Reduction				
ESL	73	2.65	1.63** (.62)	.31
Chinese and ESL	53	2.91	1.98**(.68)	.40
Non-Chinese and Non-ESL	82	−1.78	−.1.09*(.61)	.20

* < .05, ** < .01; ***6 students who identified as neither male nor female were not included in the analysis due to small sample size.

First of all, the Guide was remarkably effective for enhancing students' knowledge acquisition among all students and across all sub-groups, except for individuals who were both Chinese and ESL students. Second, the significant improvement in attitude towards stigma occurred only among non-Chinese students or among those who were both non-Chinese and non-ESL students. It is worth noting that students' attitudes towards mental health were already positive at the pre-test (M = 66.33) compared to the total score of 84 (see Table 2). The latter performed slightly better than the former. Third, the Guide was also notably effective in improving students' attitude towards seeking help across all student groups, including males, non-Chinese students, non-ESL students, those with pre-experience, and those who were both non-Chinese and non-ESL. Fourth, it is noteworthy that after the intervention, the self-reported stress level noticeably increased among ESL students or those who were both Chinese and ESL students. Conversely, a significant reduction in stress level was observed only among those who were both non-Chinese and non-ESL students. It should be noted that students’ stress was relatively low at pre-test (M = 19.74) compared to the total score of 40 (see Table 2). Additionally, no significant improvement in well-being was observed among all students or any sub-groups, which is omitted in Table 4 accordingly.

The findings mentioned above were elaborated further with respect to their respective sub-groups. First, the 103 male students obtained significant improvement in both knowledge acquisition (*p* < 0.01) and help-seeking intentions (*p* < 0.03) but not in stigma, stress level, and well-being. Conversely, only knowledge acquisition was statistically detectable (*p* < 0.01) among the 132 female students. Second, among students with Chinese heritage, knowledge acquisition was statistically detectable (*p* < 0.05). Stigma improved slightly, well-being decreased slightly, and stress increased slightly, but not significantly. Findings from non-Chinese students indicated that changes in knowledge (*p* < 0.01), stigma (*p* < 0.05), and help-seeking (*p* < 0.05) were statistically detectable; decreased stress and enhanced well-being were observed, but insignificant. Third, students without previous MHL experience obtained significant improvement in both knowledge acquisition (*p* < 0.01) and help-seeking intentions (*p* < 0.05). Knowledge improvement was also found among those with previous MHL experience (*p* < 0.01). Fourth, a crosstab analysis revealed that 81.7% of non-Chinese students fell into the non-ESL category, and 58.8% of Chinese students belonged to the non-ESL category, suggesting that ethnicity may not be the only factor that modulated the effectiveness of the intervention.

Fifth, when students were grouped into ESL and non-ESL categories to compare the five outcomes, it was found that ESL students’ stress increased significantly (*p* < 0.01). There were no significant changes with the other outcomes. For non-ESL students, both knowledge (*p* < 0.01) and help-seeking (*p* < 0.05) significantly improved, stress decreased, stigma decreased, and wellbeing improved, although not significantly. Further, among the students who are both Chinese and ESL, stress increased (*p* < 0.01); among the students of both non-Chinese and non-ESL, all of the outcomes improved significantly except for general well-being. It is noted that stress declined significantly due to the intervention, which sharply contrasted the increased-stress among ESL students regardless of their ethnicity.

Multiple regression analyses (see Table 5) showed that students’ English proficiency was a significant predictor for knowledge acquisition (*p* < 0.001). Meanwhile, ethnic background was a significant predictor for the improvement of stigma (*p* < 0.001). Gender had significant associations with changes in stigma, stress, and well-being. As reasoned in the data analysis section, the regression analyses reported in Table 5 did not include the interaction between English and ethnicity, for the regression model encompassing the interaction did not reveal any significant interaction effect on any of the five MHL variables. Furthermore, the assumption of collinearity was violated after incorporating this interaction term.

**Table 5:** Multiple Regressions for Post-Measures on Each of the Five MHL Components.

Predictors	Beta	*p*	95%CI for Beta	VIF
Post-knowledge (F(5,229) = 20.52, adjusted ${R}^{2}$=.29, *p* < .001)				
Gender	−.08	.17	[1.71,.30]	1.03
English level	−.19	< .001	[−2.93, −.69]	1.14
Ethnicity	−.05	.39	[−1.46, .57]	1.06
Pre_MHL	.09	.14	[−.25, 1.80]	1.09
Pre_ Knowledge	.44	< .001	[.61, 1.05]	1.15
Post-stigma (F(5.229) = 38.63, adjusted ${R}^{2}$=.45, *p* < .001)				
Gender	−.14	.01	[−4.85, −.69]	1.13
English level	−.09	.09	[−4.01,.30]	1.07
Ethnicity	−.16	< .001	[−5.37, −1.27]	1.10
Pre_MHL	.04	.43	[−1.20,2.79]	1.06
Pre_ Stigma	.55	< .001	[.44,.65]	1.20
Post-helpseeking (F(5,225) = 14.75, adjusted ${R}^{2}$=.23, *p* < .001)				
Gender	.06	.27	[−.51,1.79]	1.03
English	.02	.78	[−1.07,1.43]	1.07
Ethnicity level	−.10	.08	[−2.20,.13]	1.07
Pre_MHL	−.08	.17	[−1.94,.35]	1.02
Pre_ Helpseeking	.47	< .001	[.38,.63]	1.02
Post-stress (F(5,217) = 54.33, adjusted ${R}^{2}$=.55, p < .001)				
Gender	-.16	< .001	[-3.86, -.94]	1.15
English level	.10	.03	[.13,3.10]	1.07
Ethnicity	.03	.50	[-.92,1.89]	1.07
Pre_MHL	.03	.50	[-.90,1.85]	1.04
Pre_ Stress	.67	< .001	[.59,.78]	1.15
Post-wellbeing (F(5,217) = 54.33, adjusted ${R}^{2}$=.55, *p* < .001)				
Gender	.21	< .001	[1.09,3.44]	1.09
English level	−.06	.31	[−1.87,.57]	1.07
Ethnicity	−.02	.69	−1.40,.93]	1.07
Pre_MHL	.03	.56	[−.80,1.48]	1.04
Pre_ Wellbeing	.54	< .001	[.45,.68]	1.07

Gender (Female = 0, male = 1), English (Non-ESL = 0, ESL = 1), Ethnicity (Non-Chinese = 0, Chinese = 1), Pre_MHL (Not having prior experience = 0, having prior experience = 1).

Table 6 quantitatively demonstrates the disparity between different groups of students according to their MHL outcomes. Specifically, non-ESL students performed better than ESL students in knowledge acquisition, and the gap between them was statistically detectable. The relationship between English proficiency and stress level change among participants favoured non-ESL students. Similarly, students without Chinese heritage demonstrated improved attitudes toward both stigma and help-seeking following the intervention. We can also learn from Table 6 that, on average, female students did significantly better than the males in obtaining positive attitudes towards mental health and gained more knowledge, although not significantly; the male students performed significantly better than the females in reducing stress and enhancing well-being. Meanwhile, males demonstrated more willingness than females to seek help, although the gap was not statistically detectable.

**Table 6:** Means of the Post-Test MHL Outcomes by Gender, Ethnicity, and English After Controlling for Both Pre-Measures in ANCOVA.

Post-measures	Chinese	Non-Chinese	ESL	Non-ESL	Male	Female
Knowledge	15.68	16.55	14.62	17.04	15.74	16.32
Stigma	64.93	70.14	64.08	68.27	63.77	69.40
Help-seeking	25.38	26.78	26.83	25.90	26.35	25.73
Stress	19.78	19.57	20.06	19.51	16.39	22.35
Wellbeing	17.67	17.88	17.33	17.84	19.75	16.10
	Both Chinese and ESL			Both Non-Chinese and Non-ESL		
Knowledge	13.72			17.05		
Stigma	61.98			70.30		
Help-seeking	25.83			26.67		
Stress	20.06			19.17		
Wellbeing	17.40			18.08		

Finally, the five two-way ANCOVA models revealed no interaction effects between ethnicity and English proficiency on the five MHL variables. This finding is consistent with what was observed in the multiple regression models, where the interaction between English proficiency and ethnicity was insignificant. Notably, Levene's tests detected a violation of the assumption of homogeneity of variances in two of the five models. This indicates that the third hypothesis in the present study is either rejected or lacks valid justification.

## Discussion

The analysis showed that significant improvements were found in knowledge and help-seeking intentions among all students involved, and stigma change was relatively minor. This is consistent with what has been found in many MHL interventions^6,8^. Our observation is that student attitudes towards mental health were already very positive (indicating lower stigma), and stress levels were relatively low at baseline, suggesting a potential ceiling effect for improvement.

Past research demonstrates that effectiveness of an intervention depends not only on how it is designed and implemented (its mechanism), but also on contextual factors such as age, gender/sex, ethnicity, and English proficiency^3,9,18^. We found male students achieved significant gains of knowledge and help-seeking intentions, while female students showed significant gains only in knowledge acquisition, which is consistent with similar school-based studies^11^. No significant improvements were found in stigma, stress, or well-being among both male and female students. Previous studies identify inconsistent gender differences in MHL outcomes^12,14^. For instance, Wong et al.^14^ demonstrated that female participants believed medications to be relatively more harmful compared to male counterparts, whereas Wang et al.^15^ found no gender differences on help-seeking behaviors, stigma, or knowledge among Asian-American high school students. However, these cross-sectional studies lacked long-term observations.

By using a longitudinal design, the present study found that, except for help-seeking, both male and female students improved their MHL and general health outcomes at a similar pace (Table 4). Furthermore, multiple regression models revealed that gender was the most significant predictor of change among three attitudinal/emotion-related MHL outcomes (stigma, stress, and well-being); however, this had little to do with knowledge acquisition (Tables 5 and 6). This is an important finding given that the role of gender in the effectiveness of school-based MHL interventions was not reported in available systematic reviews on this topic^8,33–37^.

The impact of students’ ethnicity on the effectiveness of the intervention was also investigated. Only knowledge improvement was statistically detectable in the students with Chinese heritage. In contrast, non-Chinese students demonstrated statistically detectable improvements in knowledge, stigma, and help-seeking. As such, in order to thoroughly assess the effectiveness of a school-based MHL intervention in multicultural contexts, participants’ ethnicity needs to be considered. This aligns with the finding that the students with Chinese heritage in Canada exhibit different attitudes toward seeking treatment for social anxiety compared to those with European heritage^26^. Additionally, Li et al.^16^ found that among acculturation, ethnicity, and English proficiency, Chinese ethnicity was the only factor negatively associated with help-seeking attitudes among Chinese international students in the US. More information is needed to further investigate why students with Chinese heritage performed differently than non-Chinese counterparts.

We found that ESL students achieved no significant improvement in stigma, help-seeking, or well-being, but rather demonstrated significantly increased stress at post-test (Table 4). Their mental health knowledge was the same as Chinese students with significant gains (Tables 4 and 6). This may suggest that both ethnicity and English proficiency impacted some of the intervention outcomes; however, English proficiency exhibited more influence than ethnicity, particularly in the case of knowledge acquisition where changes were solely predicted by English proficiency (Table 5). Moreover, findings from the multiple regression analysis suggest that a lack of English proficiency during the intervention may have increased the stress levels of ESL students (Table 4). This may inform schools to adapt the intervention and its delivery to accommodate ESL students; perhaps by using lay English, or by relying less on language-dependent learning styles and pedagogies (e.g., audio and visual teaching methods, contextual clues, English-only scaffolding opportunities). The analysis further indicated that non-Chinese students performed similarly to non-ESL students in improving MHL outcomes. The similarity may be due to a large overlap effect; almost 82% of non-Chinese students were non-ESL students. Overall, the intervention was most effective among students who were both non-Chinese and non-ESL and was least effective among those who were both Chinese and ESL (Tables 4 and 6).

The impact of students’ language proficiencies on mental health knowledge acquisition warrants further investigation. Contemporary theories of learning claim that learning involves acquisition of knowledge, attitudinal change, and shifts in belief, emotion, and behavior. MHL interventions can be viewed as learning processes that instigate and measure changes of knowledge, attitudes, emotions, and behaviors^3,39^. One’s cognition is socially constructed and context-dependent^20^, and language plays a fundamental role in our cognitive development, knowledge acquisition, thinking, emotions, behaviors, and interactions with others^40^. When assessing the effectiveness of MHL interventions, English proficiency, as a social-cognitive factor, can provide powerful insight into MHL outcomes. The present study is the first attempt to examine MHL interventions from social cognitive theories of learning.

The present data show that, after controlling for previous MHL experience, English proficiency was the sole significant predictor for students’ knowledge acquisition, whereas ethnic background was the only significant predictor for the change in the attitude-related MHL outcomes. As stated, attitude is a multifaceted construct that may be more resistant to change than forms of knowledge^41^, which could help explain why stigma and help-seeking intentions changed relatively less than knowledge. This also suggests that change in mental health knowledge may be a prerequisite of the changes in attitude-related components of MHL considering the theory about cognitive influences on attitude^42^. Presumably, delayed effects on attitudinal and behavioral changes could exist and be worthy of future exploration. This actually elicits the necessity for future research to explore potential interactions between knowledge acquisition and attitudinal and behavioral changes in MHL, providing a rationale for investigating the wide-ranging interactions among MHL components.

The above finding aligns with the observation that the students with Chinese heritage in Canada exhibit different attitudes toward seeking treatment for social anxiety compared to those with European heritage^26^. This highlights the considerable influence of one's cultural background on their attitude toward seeking mental health services. Additionally, a positive association was found between the level of acculturation to American society and the willingness to seek professional help^5^. These findings collectively suggest that acculturation can serve as a strategic approach to facilitate attitude change among Chinese students. Acculturation refers to a dual process, encompassing cultural and psychological transformations resulting from the interaction between individuals and two or more cultural groups^46^. This definition highlights the vital role of interpersonal communication capabilities in acculturation and underscores the crucial importance of English proficiency in the acculturation process within English-speaking societies. Consequently, it can be inferred that students' English proficiency is significantly and directly associated with their acquisition of MHL knowledge. Furthermore, it may exert an indirect influence on changes in attitude-related MHL like help-seeking through the process of acculturation. As stated in the introduction section, the variation in attitudes toward seeking professional services among Chinese individuals is primarily attributed to differences in English proficiency^28^. These findings underscore the significance of English proficiency and acculturation in influencing attitude-related MHL components, such as stigma and help-seeking, among Chinese students. Additionally, according to Radez et al.^36^, knowledge is one of the main barriers of seeking professional help among general population.

Taken together, it turns out that, in addition to conventional MHL interventions, fostering positive help-seeking attitudes among Chinese students elicits close collaboration between mental health professionals, such as school counselors and school psychologists, and educational professionals like curriculum developers, instructional designers, and classroom teachers. This collaboration aims to enhance MHL knowledge, improve English proficiency, and support better acculturation into mainstream societies. Hence, further research is essential to investigate the effective implementation of this interdisciplinary commitment. The aim is to design MHL programs tailored to both ethnic and English language needs for Chinese students who lack adequate English proficiency.

Our analyses found no significant interaction effects of ethnicity and English proficiency on MHL and general health, which rejected our third hypothesis. Regardless, MHL intervention designers and practitioners should consider students’ ethnicity and English proficiency when designing, implementing, and assessing school-based MHL interventions in multicultural and multilingual settings. Mansfield et al.^43^ advised that adolescent MHL research should develop more theoretically and psychometrically reliable, valid and feasible measures that better suit adolescents’ cognitive development. Given that language as a social-cognitive tool plays a central role in development and learning^44^, it should be considered an important variable by researchers and practitioners. English proficiency is malleable and can improve, which may lead to improved MHL outcomes and enhanced effectiveness of school-based MHL interventions.

Finally, our findings shed light on issues that may be worth further inquiry. First, as an evolving concept, the widely accepted MHL definition is multi-faceted and embraces various constructs: cognition, attitudes, emotions, and behaviors^3^. Spiker and Hammer^40^ called for reconceptualizing MHL as a theory containing interrelated and independent constructs, rather than a single construct containing different dimensions. Following this logic, Mansfield et al.^43^ proposed that a multi-construct theory of MHL may help us understand ways in which MHL constructs relate, enabling the formation of better integrated theories to improve adolescent mental health. Our study suggests further empirical and theoretical investigation of interrelationships among MHL constructs. This is particularly relevant as our earlier discussion indicated that students' acquisition of MHL knowledge likely serves as a prerequisite for the transformation of attitude-related MHL components, including help-seeking behavior and stigma perception.

Additionally, the present study applied social-cognitive theories of learning to a school-based MHL intervention that reconceptualized English proficiency as a social-cognitive factor underlying the effectiveness of the intervention. Considering the theoretical rationale of our hypotheses and analysis of distinctions between Chinese and non-Chinese Canadian students, future research should also involve other ethnic minority groups to test the generalizability of these hypotheses.

One limitation of the study must be noted. English proficiency was measured by students’ subjective self-reports and was coded as a binary variable. Compared to continuous measures, dichotomous measures are more likely to lose information; it is difficult to measure and disseminate nuance from such responses^45^. Moreover, students’ English proficiency is indirectly inferred from their self-reported categories of either ESL or non-ESL, rather than by any standardized assessment like IELTS. Follow-up research may use standardized assessments of English proficiency as a continuous variable. Another limitation is that all the participants were from the same grade, making it difficult to ascertain how students’ developmental stage, personality, and socialization could impact effectiveness of the intervention. Finally, MHL is defined as: understanding how to obtain and maintain positive mental health; understanding mental disorders and their treatments; decreasing stigma related to mental disorders; and, enhancing help-seeking efficacy. However, the measurement of positive mental health that we was missing in the study. We will address this by using measures such as resilience scales in future studies as the goal of our target intervention is to help youth become more resilient in dealing with mental health.

## Conclusions

The Guide was effective in significantly improving mental health knowledge and help-seeking intentions for all students involved, regardless of demographic characteristics. It was most effective among both non-Chinese and non-ESL students, who achieved significant improvements in knowledge, stigma, help-seeking, and stress. English proficiency and ethnicity function differently in improving MHL outcomes.

## Data Availability

Data is available upon request to the corresponding author Dr. Yifeng Wei.
